# Adenopathies: A Confusing Presentation of Primary Biliary Cholangitis

**DOI:** 10.7759/cureus.33814

**Published:** 2023-01-16

**Authors:** Catarina Maciel, Daniela Augusto, Nadia Tenreiro, Filipe Martins

**Affiliations:** 1 Internal Medicine, Centro Hospitalar Trás-Os-Montes e Alto Douro (CHTMAD), Vila Real, PRT; 2 General Surgery, Centro Hospitalar Trás-Os-Montes e Alto Douro (CHTMAD), Vila Real, PRT

**Keywords:** asthenia, pruritus, lymphadenopathy, adenopathy, primary biliary cholangitis

## Abstract

Primary biliary cholangitis (PBC) is an autoimmune disease in which the intrahepatic bile ducts are destroyed. Its symptoms include chronic fatigue, pruritus, steatorrhea, and jaundice, with variable clinical course. We are introducing a case of a 65-year-old woman with anorexia, weight loss, asthenia, and pruritus. The imaging studies revealed dilatation of the intrahepatic and common bile ducts and adenopathies at the level of the hepatoduodenal ligament, histologically compatible with reactive lymphadenitis. After the exclusion of neoplasia, she was referred to Internal Medicine where positivity was obtained for anti-mitochondrial autoantibodies suggestive of PBC. After the initiation of therapy, there was a resolution of the clinical symptoms and the adenopathies were no longer detected in subsequent studies. The authors intend to highlight this case, especially due to the presence of adenopathies and constitutional symptoms where PBC should also be considered, as a differential diagnosis.

## Introduction

Primary biliary cholangitis (PBC) is an autoimmune disease that causes the destruction of intrahepatic bile ducts, predominantly in middle-aged women. The predominant symptoms are chronic fatigue, intense pruritus, steatorrhea, and jaundice, which may evolve into cirrhosis. Diagnosis is made by clinical, biochemical (elevation of alkaline phosphatase and/or gamma-glutamyltransferase), autoimmune markers - positivity for anti-mitochondrial antibodies (AMA) - or by liver biopsy in AMA-negative patients [1 -2]. PBC can be linked to reactive lymphadenopathy, with prevalence reported between 27% and 88% in the larger series of patients with PBC. These findings imply the importance of vast diagnostic approach work-up to exclude differential diagnosis [3]. The mechanism and clinical relevance of these adenopathies remain to be clarified.

This case was displayed as a poster at the 20th European Congress of Internal Medicine in June 2022.

## Case presentation

In 2018 a 65-year-old woman, independent, was referred to the General Surgery outpatient clinic for epigastric pain, weight loss, and post-cholecystectomy bile duct dilatation. In 2012, she has already had a biliary mild acute pancreatitis for which she underwent laparoscopic cholecystectomy. In 2017 a bone densitometry scan had already determined she had osteopenia. She did not take any chronic medication though.

For the past year, she reported epigastric pain linked to anorexia. She lost six kgs in six months and displayed debilitating asthenia. Given the constitutional symptoms, an extensive etiological study including upper and lower endoscopy was carried out. It is intended to rule out gastrointestinal malignancy. The abdominal computed tomography (CT) revealed enlarged globular liver, without signs of chronic liver disease and slight dilatation of the intrahepatic and common bile ducts (10mm). The study also revealed the presence of adenopathies. The largest measured 1.6cm by short axis in portocaval space of the hepatoduodenal ligament, with moderate fixation in positron emission tomography (PET).

Due to potentially retained bile duct stones, magnetic resonance cholangiopancreatography (MRCP) was also performed which confirmed biliary duct dilatation, without obstruction but with contrast uptake in the periampullary bile duct. We obtained tumor mark panels where Carbohydrate antigen 19-9 (Ca 19.9) was mildly elevated. So we performed a percutaneous biopsy with aspiration cytology of hepatoduodenal ligament adenopathy, which revealed nonspecific reactive lymphadenitis. In January 2019 the patient underwent endoscopic retrograde cholangiopancreatography (ERCP) with sphincterotomy and biopsy of the ampullary region. This led to complications with pancreatitis post-ERCP, managed in an inpatient setting. Histological examination of the ampullary region ruled out dysplastic lesions or neoplastic involvement. 

After the extensive study and since there was no clear evidence of an active gastrointestinal or biliary malignancy, she was referred to Internal Medicine. The patient confirmed severe epigastric pain, as well as nausea and early fullness which - combined - led to a decrease in food intake. When asked, she also referred to debilitating symptoms such as intense itching and severe asthenia, which appeared at the same time as the other symptoms. She denied fever or night sweats, intestinal tract symptoms, including steatorrhea, or jaundice. Autoimmune symptoms such as joint complaints, *sicca* symptoms, skin or ocular changes, or aphthous eruptions, as well as cardiovascular, respiratory, or genitourinary symptoms were non-existent. Upon physical examination, the body mass index was of 22.8kg/m^2^ (body mass 52 kg and height 1.51 m). She presented skin erosion lesions compatible with the described pruritus. She had normal cardiopulmonary auscultation, soft and depressible abdomen, without defense or pain, without masses or organomegaly, palpable lymphadenopathy, stigmata of chronic liver disease, or peripheral edema.

After an initial evaluation, we considered the following differential diagnoses: autoimmune liver diseases or biliary tracts such as PBC, viral infection, or less common inflammatory conditions such as IgG_4_ cholangiopathy or sarcoidosis. Table 1 presents the laboratory findings obtained at different moments during the patient's evaluation.

**Table 1:** Laboratory evaluation since the beginning of the patient's surveillance and after the diagnosis of PBC (2018 – 2022). We emphasize the improvement of cholestasis after the introduction of targeted therapy, as well as the altered tumor marker (Ca 19.9), which normalized after the beginning of the same. Note that, at 04/2019, the patient started the ursodeoxycholic acid (15mg/kg/day). AST: Aspartate aminotransferase; ALT: alanine aminotransferase; FA: alkaline phosphatase; gGT: gamma-glutamyltranspeptidase; BT – total bilirubin; BD – direct bilirubin; CEA: carcinoembryonic antigen.

Analytical parameter	Date of laboratory evaluation (month/year)					
	09/2018	02/2019	04/2019	07/2019	06/2020	06/2022
AST (U/L) (<35)	32	34	41	28	25	29
ALT (U/L) (<33)	28	23	35	22	19	19
FA (U/L) (7-32)	95	170	194	12	10	14
gGT (U/L) (5-105)	159	205	108	67	58	73
BT (mg/dL) (<1.2)	0.4	0.4	0.5	0.5	0.4	0.4
BD (mg/dL) (<0.3)	0.1	0.1	0.2	0.2	0.2	0.1
Ca 19.9 (u/mL) (0-37)	52	41				28
α-fetoprotein (UI/mL) (1-8)				0.8	0.8	0.8
CEA (mg/dL) (0-31)		1.8				
Ca 15.3 (U/mL) (0-31)		20				
Ca 125 (U/mL) (0-35)		39				20

**Figure 1:**
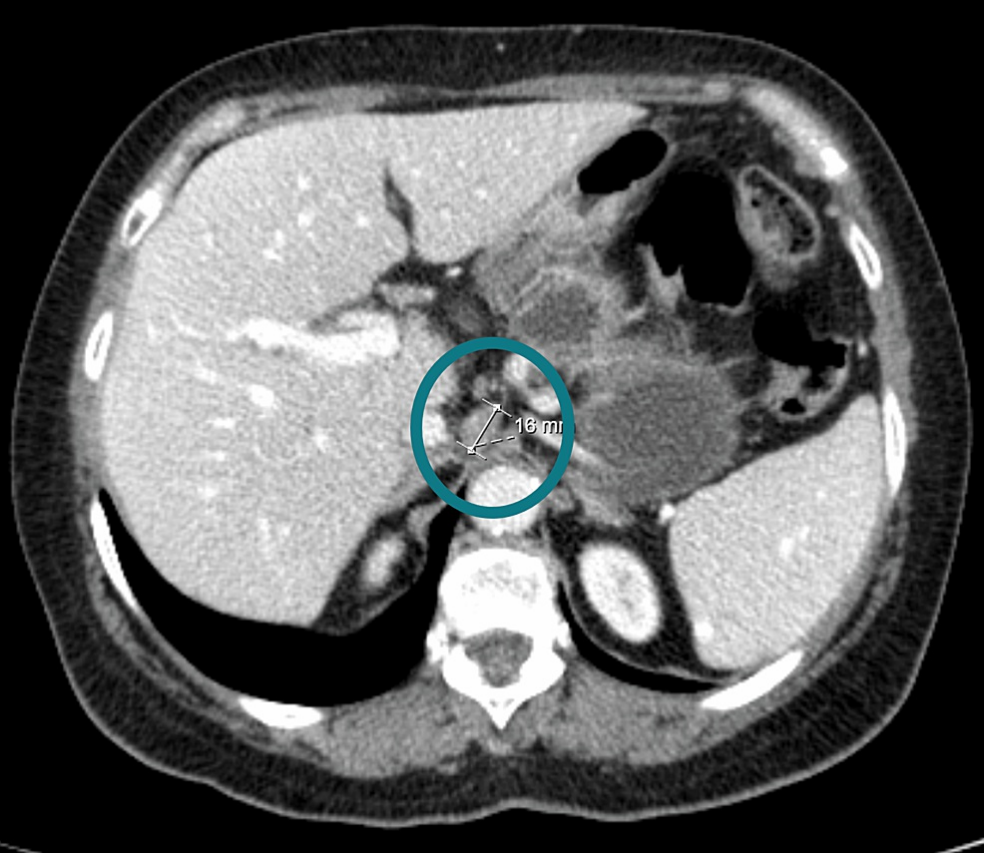
Abdominal CT from 2018, before the diagnosis, where intercavalportal adenopathies were identified. (Circle)

**Figure 2:**
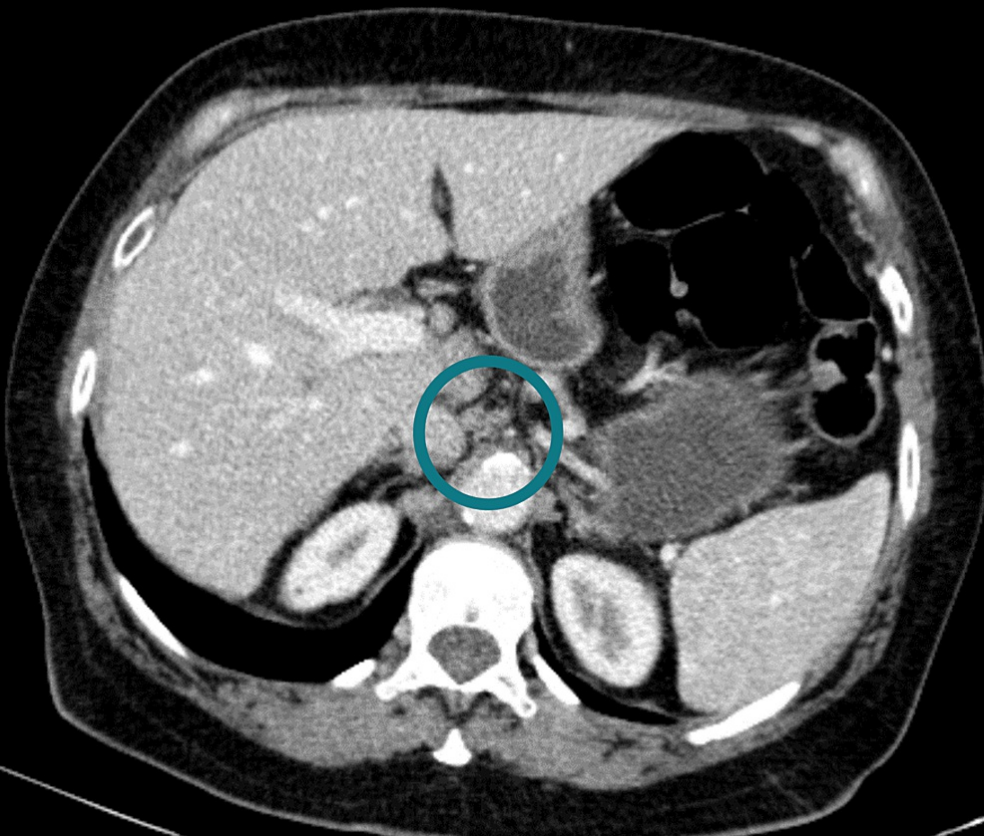
Abdominal CT from 2020, after treatment with ursodeoxycholic acid and without enlargement of ganglia.

Complementary diagnostic tests revealed an increase in the sedimentation velocity of 67mm/1^st^h, but the remaining inflammatory parameters were normal. In liver biochemistry, the pattern of cytocholestasis with normal bilirubin was maintained. The directed autoimmune study showed strong positivity for antinuclear and anti-mitochondrial autoantibodies, with anti-M2 greater than 200 and positive antiSP-100. The remaining laboratory/biochemistry studies were unremarkable: normal complete blood count, peripheral smear, coagulation study, renal function, ionogram, lipid profile, vitamin levels, and thyroid function remained unchanged, negative serology for HIV, hepatotropic viruses and syphilis, without elevation of angiotensin-converting enzyme (ACE) and without complement consumption. In view of the findings of pruritus, asthenia, weight loss, osteopenia, hepatic cholestasis, hepatoduodenal ligament adenopathies, and positive AMA, PBC was assumed as a definite diagnosis and therapy with ursodeoxycholic acid was initiated at 15mg/kg/day. Follow-up exams further to 3 months of therapy showed resolution of symptoms and normalization of liver biochemistry (Table 1). Adenopathy size reduction was documented on a one-year follow-up CT scan (Figures 1, 2).

## Discussion

Amongst the most common findings in individuals with PBC, the patient reported severe asthenia and significant weight loss that led to the need for rapid exclusion of malignancy. With this case, we intend to demonstrate that PBC can, sometimes, display adenopathies and exuberant constitutional symptoms, which can be mistaken for malignancy. An extensive investigation was initially performed to rule out that diagnosis. Abdominal adenopathies are not uncommon in autoimmune liver diseases, particularly in PBC. The authors also consider those adenopathies could be the result of previous pancreatitis that the patient developed. However, comparing previous exams, we confirmed they weren't present and the time interval allows us to define with greater certainty the causality between PBC and adenopathies. When retrospectively evaluating abdominal CT scans of 21 patients with PBC [4], the authors looked for adenopathies that were evident in 81% of patients in a periportal location and in the gastrohepatic ligament. These matched histologically to reactive hyperplasia.

Another series of 283 patients with PBC and evaluation by magnetic resonance [5], showed the presence of adenopathies in 78.1% of the cases. However, there are also studies that demonstrate lower prevalences of this finding [3][6]. In our institution, we retrospectively evaluated 48 cases diagnosed with PBC, monitored between 2000 and 2019. 68.7% (n:33) of these patients underwent CT and of these 15.2% (n:5) had adenopathy. In three cases, they resolved after the introduction of therapy, and in the remaining cases, they were linked to other pathologies (sarcoidosis, myelodysplastic syndrome, and Hodgkin's lymphoma).

In our case, once therapy started, there was the resolution of pruritus and improvement of asthenia and anorexia, as well as normalization of inflammatory parameters and liver biochemistry. In the serial imaging evaluation, it was verified that the initially present adenopathies disappeared after the start of therapy (Figure 1, 2). Given the biliary dyskinesia associated with the inflammatory process of PBC and the post-cholecystectomy status, we considered that the symptoms of early fullness and anorexia could be the result of these findings, corroborated by the clinical improvement after starting therapy. We also emphasize the normalization of Ca 19.9 (a marker produced by an inflamed biliary tree) after the introduction of ursodeoxycholic acid, during patient surveillance. Since neoplasia was the differential diagnosis that the authors thought in this clinical case, when elevated, it was relevant to exclude the possible existence of neoplasia in a patient with important constitutional symptoms. However, it is important to highlight that, like other tumor markers, Ca 19.9 is suggestive of neoplasia in situations of gastric cancer, pancreatic cancer, gallbladder cancer, or cholangiocarcinoma, but high levels can also be a sign of other types of cancer or certain conditions as gallstones, cirrhosis or liver inflammation. The patient maintains follow-up at an outpatient clinic with no evidence of disease progression after two years with no need for any other subsequent therapy. 

## Conclusions

Due to the association with the appearance of neoplasms, constitutional symptoms imply a detailed multisystemic evaluation, especially if associated with adenopathies, given that the time for diagnosis and initiation of therapy has an implication on the progression of an oncological disease. However, diseases with an inflammatory and/or autoimmune substrate may also have this presentation and, therefore, be one of the plausible differential diagnoses. As presented in this article, PBC can show adenopathies, resulting from the hyperinflammation underlying the disease. We intend to highlight this case so that, through a suggestive clinical context, this pathology can be investigated. PBC has a variable clinical course, in which clinical improvement is achieved after targeted therapy in the overwhelming majority of patients, as we saw in this case. Due to the risk of progression to cirrhosis, its diagnosis must be equated and clinical and analytical monitoring of the disease ensured.
